# Sequential Advancement Method: A Funnel-Based Technique for Safe Placement of Small Breast Implants

**DOI:** 10.1093/asjof/ojag142

**Published:** 2026-07-04

**Authors:** Claire G Olivas, Orr Shauly, Daniel J Gould

## Abstract

Recent aesthetic trends in breast augmentation favor smaller implants and minimal-incision techniques. However, placement of very small implants (100-165 cc) may pose unique mechanical challenges during funnel-assisted insertion because of the smaller contact surface area available for force distribution. The Sequential Advancement Method (SAM) provides a simple and reproducible solution by placing a larger sizer behind the implant within a sterile funnel-based implant delivery device to distribute force more evenly. This technique enables safe prepectoral placement through 2 cm incisions, reducing peak pressure and lowering tissue trauma. Although larger cohort studies are needed to validate outcomes, SAM offers a biomechanically sound strategy to support patient aesthetic preferences while decreasing the risk of implant-related complications.

Level of Evidence: 5 (Therapeutic)

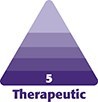

Recent aesthetic trends in breast augmentation have shifted toward natural, athletic, and proportionate contours, reflecting a broader movement away from dramatic volume enhancement, also known as the “era of the great deflation.”^1^ Both patients and surgeons increasingly prioritize balanced results that compliment active lifestyles and lean body frames.^2^ This trend is mirrored in global surgical data, with breast implant removal procedures nearly doubling between 2017 and 2022, surpassing over 320,000 explants annually.^3^ In parallel to these trends, there has been growing popularity of minimal-incision breast augmentation techniques, such as the funnel-based implant delivery device approach and subfascial techniques.^4^ These techniques are associated with the use of smaller implants and shorter incisions. However, the placement of very small implants (100-165 cc) may pose unique mechanical challenges during funnel-assisted insertion because of the smaller contact surface area available for force distribution.

Because of the challenges posed by these smaller implants, surgeons have been seeking to optimize outcomes through alternative methods. One of these methods is the Sequential Advancement Method (SAM), which provides a more biomechanically sound, reproducible option for surgeons. The purpose of this report is to describe the operative steps and biomechanical rationale underlying the SAM for insertion of very small implants through short-incision augmentation. The technique is demonstrated stepwise by the senior author (D.J.G.) in Video, with key operative stages illustrated in Figure 1.

**Figure 1:**
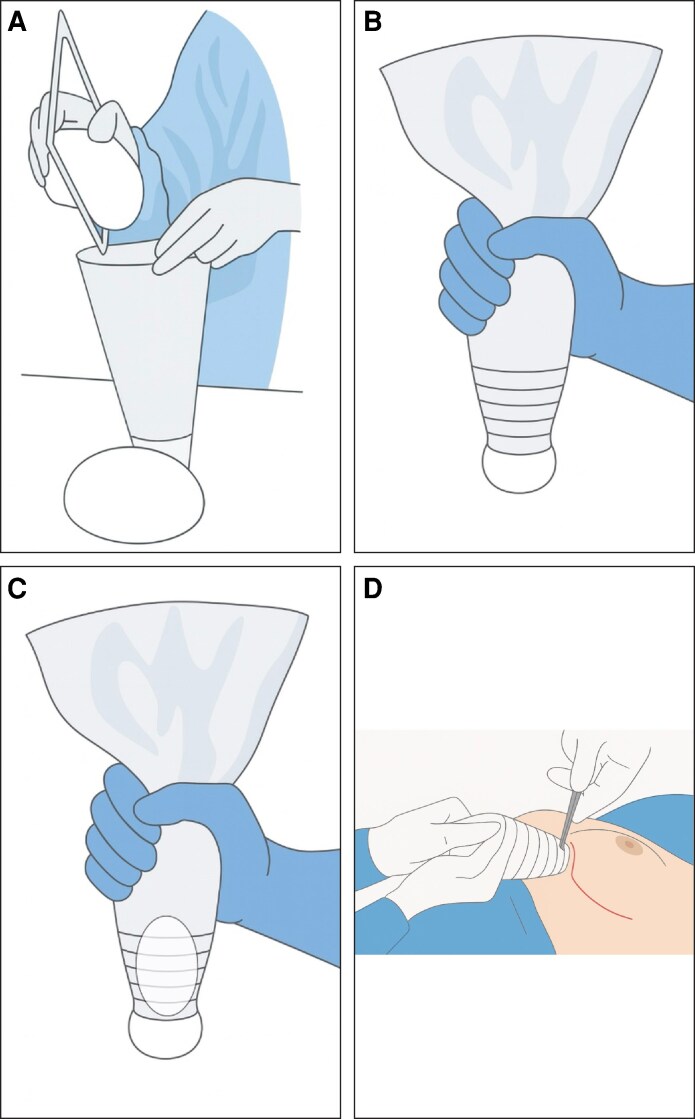
(A) Funnel preparation with implant immersion in a sterile solution. (B) Placement of a small implant using no-touch delivery. (C) Advancement with a larger saline sizer positioned behind the implant. (D) Funnel insertion and delivery into the prepectoral pocket. The authors used ChatGPT 5.5 (OpenAI, San Francisco, CA) to create these images. After using this tool, the authors reviewed and edited the content as needed and take full responsibility for the content of the publication.

## STEP 1: FUNNEL PREPARATION

A sterile funnel-based implant delivery device, such as the Preservé or Keller-style funnel, is prepared, starting with trimming the distal end of the funnel using a sterile straight blade, immersing the funnel in a sterile solution, and then loading the small implant (100-165 cc) into the large end of the funnel using the no-touch delivery technique.^5^

## STEP 2: SEQUENTIAL ADVANCEMENT SETUP

To optimize the biomechanics of the maneuver, a larger saline sizer (typically 250-350 cc, although the exact size is not critical) is placed behind the implant within the funnel. The presence of the larger sizer provides a broader contact surface, allowing a forward force to be distributed evenly across the implant during advancement.

## STEP 3: SEQUENTIAL ADVANCEMENT MANEUVER

The funnel is inserted approximately 1 cm into the 2 cm incision. Compared with the standard inframammary approach, the incision is shifted approximately 1.5 cm laterally and oriented obliquely or vertically toward the prepectoral plane rather than horizontally. Steady downward pressure is then applied from the proximal end to advance the implant into the prepectoral pocket without direct skin or glove contact, reducing contamination risk.^6,7^ Following delivery of the definitive implant, the sizer remains within the funnel and is removed from the operative field together with the funnel.

## STEP 4: POCKET CONSIDERATIONS

Using the funnel in a prepectoral plane provides a compliant pocket with minimal resistance, ensuring atraumatic and hemostatic insertion. A carefully performed tunnel dissection directs the implant into the desired position while preventing inferior displacement, or “bottoming out,” a complication often encountered with conventional pocket dissection.^8^ The sequential advancement maneuver prevents overdissection of the pocket, so the implant is immobile and thus unlikely to displace inferiorly over time.

## DISCUSSION

The biomechanical rationale underlying the SAM relates to the redistribution of insertional forces during funnel-assisted implant advancement. In very small implants, the reduced proximal contact interface within the funnel may result in greater concentration of focal stress during advancement through short incisions, particularly within tighter surgical planes. By positioning a larger saline sizer posterior to the implant within the funnel, the SAM increases the proximal contact surface area, allowing insertional forces to be distributed more uniformly during delivery. This broader force distribution may reduce localized shell stress and improve surgeon mechanical advantage during implant advancement.

This concept is consistent with established mechanical principles describing force transmission across curved surfaces. As described by Mayhew, increasing the effective contact surface area may facilitate more even distribution of applied forces during advancement.^9^ Accordingly, the larger proximal interface created by the saline sizer may reduce focal stress concentration on the implant shell while improving controlled advancement of the implant through short-incision approaches.

In addition to facilitating more controlled implant delivery, the technique may also improve ergonomics for surgeons with smaller hands by decreasing localized resistance during insertion.

The SAM was developed through iterative operative refinement by the senior author (D.J.G.) to address the technical challenges associated with funnel-assisted insertion of very small implants. Early experience suggested that insertion through the submuscular plane generated substantial resistance during advancement, particularly with implants in the 120 to 140 cc range. Transitioning to prepectoral placement provided a more compliant insertion pathway and facilitated more controlled implant delivery through short incisions. Over time, the maneuver evolved to incorporate the use of a larger saline sizer positioned posterior to the implant within the funnel to improve force distribution during advancement. For teaching purposes, however, the technique can be effectively demonstrated in-office using a discarded funnel and sizer, which offers a practical way to illustrate the maneuver to trainees.

The SAM is currently limited to implants that are compatible with funnel-based delivery systems. Although the Keller funnel and similar devices are widely available, this technique may not be feasible in settings where such tools are unavailable or with implant types that are not designed for no-touch insertion. Although this technique has demonstrated technical feasibility in the senior author's experience, further biomechanical and prospective clinical evaluation is needed to better characterize the safety, reproducibility, and broader applicability of the technique.

Future investigation should include bench-top mechanical testing to quantify insertional forces and force distribution during funnel-assisted delivery with and without the SAM. Such data would provide evidence for the proposed reduction in peak pressure and implant rupture risk. Beyond laboratory validation, multisurgeon adoption studies will be critical to determine whether the technique is reproducible across different surgical styles, levels of experience, and practice settings. Comparative trials with conventional subglandular, subfascial, and submuscular placements may also clarify patient-specific advantages and help define ideal indications for sequential advancement. Ultimately, broader adoption will depend on both demonstrable clinical benefit and ergonomic advantages.

## CONCLUSIONS

The SAM represents a simple, reproducible, and biomechanically rational approach for the safe placement of very small implants through incisions as short as 2 cm. By distributing insertional forces and reducing peak pressure, the technique minimizes the risk of implant rupture while enhancing surgeon ergonomics. In addition, its reliance on prepectoral or subfascial positioning aligns with emerging breast preservation strategies and underscores the advantages of funnel-assisted, atraumatic, no-touch implant delivery. With further validation in larger cohorts and across diverse surgical practices, this method has the potential to support broader adoption of funnel-assisted, muscle-preserving breast augmentation.
